# Effect of the Pregnant+ Smartphone App on the Dietary Behavior of Women With Gestational Diabetes Mellitus: Secondary Analysis of a Randomized Controlled Trial

**DOI:** 10.2196/18614

**Published:** 2020-11-04

**Authors:** Lisa Garnweidner-Holme, Lena Henriksen, Liv Elin Torheim, Mirjam Lukasse

**Affiliations:** 1 OsloMet - Oslo Metropolitan University of Applied Sciences Oslo Norway; 2 Faculty of Heath and Social Sciences University of South-Eastern Norway Campus Vestfold Norway

**Keywords:** gestational diabetes mellitus, diet, mHealth, mobile phone, randomized controlled trial

## Abstract

**Background:**

The prevalence of gestational diabetes mellitus (GDM) is increasing worldwide. A healthy diet and stable blood glucose levels during pregnancy can prevent adverse health outcomes for the mother and the newborn child. Mobile health may be a useful supplement to prenatal care, providing women with targeted dietary information concerning GDM.

**Objective:**

We analyzed secondary data from a two-arm, multicentered, nonblinded randomized controlled trial to determine if a smartphone app with targeted dietary information and blood glucose monitoring had an effect on the dietary behavior of women with GDM.

**Methods:**

Women with a 2-hour oral glucose tolerance test level of ≥9 mmol/L were individually randomized to either the intervention group receiving the Pregnant+ app and usual care or the control group receiving usual care only. Eligible women were enrolled from 5 diabetes outpatient clinics in the Oslo region, Norway, between October 2015 and April 2017. The Pregnant+ app promoted 10 GDM-specific dietary recommendations. A healthy dietary score for Pregnant+ (HDS-P+) was constructed from a 41-item food frequency questionnaire and used to assess the intervention effect on the dietary behavior completed at trial entry and at around gestation week 36. Dietary changes from baseline to week 36 were examined by a paired sample two-tailed *t* test. Between-group dietary differences after the intervention were estimated with analysis of covariance, with adjustment for baseline diet.

**Results:**

A total of 238 women participated: 115 were allocated to the intervention group and 123 to the control group. Of the 238 women, 193 (81.1%) completed the food frequency questionnaire both at baseline and around gestational week 36. All the participants showed improvements in their HDS-P+ from baseline. However, the Pregnant+ app did not have a significant effect on their HDS-P+. The control group reported a higher weekly frequency of choosing fish meals (*P*=.05). No other significant differences were found between the intervention and control groups. There were no significant demographic baseline differences between the groups, except that more women in the intervention group had a non-Norwegian language as their first language (61 vs 46; *P*=.02).

**Conclusions:**

Our findings do not support the supplementation of face-to-face follow-up of women with GDM with a smartphone app in the presence of high-standard usual care, as the Pregnant+ app did not have a beneficial effect on pregnant women’s diet.

**Trial Registration:**

ClinicalTrials.gov NCT02588729; https://clinicaltrials.gov/ct2/show/NCT02588729

## Introduction

Gestational diabetes mellitus (GDM) is defined as hyperglycemia detected at any time during pregnancy [1]. The prevalence of GDM is increasing worldwide and ranges from 1% to 20% globally depending on the screening procedure and population characteristics [2]. The prevalence of GDM in Norway was 5% in 2018, according to the Norwegian Medical Birth Registry [3]. However, a cohort study in a district in Oslo identified GDM in 13% of all women, 11% of ethnic Norwegians, and 12%-17% of women in groups of non-European origin [4]. Women of South Asian and African origins tend to develop GDM at a lower body mass index and age compared to White Europeans [5]. The other risk factors for developing GDM include overweight and obesity, advanced maternal age, a family history of diabetes, and GDM in a previous pregnancy [6]. Even though GDM resolves in most women after delivery, its development may affect the health of both mothers and children in the short and long terms [7,8].

A healthy diet and stable blood glucose levels throughout pregnancy can prevent adverse health outcomes for the mother and the newborn child [9]. About 85% of the women diagnosed with GDM can manage the disease with lifestyle changes such as healthy eating and physical activity, without the need for oral metformin or insulin therapy [10]. However, lifestyle changes presuppose knowledge, motivation, and follow-up by health care professionals [11]. Pregnant women are often in contact with health care professionals; however, perinatal care involves dealing with many health-related issues, and there are some indications that women are not provided sufficient information about the management of GDM by their health care professionals [12,13].

Mobile health (mHealth)—defined as medical and public health practice supported by mobile devices such as smartphones, patient monitoring devices, personal digital assistants, and other wireless devices [14]—may be a useful supplement to perinatal care by providing women with GDM with dietary information and the opportunity to register blood glucose levels [15]. A scoping review has found several ongoing randomized controlled trials (RCTs) that evaluate the effectiveness of smartphone apps in the management of GDM [16]. Some results of these RCTs have been published recently [17,18]. Even though these studies did not find any effect on the glycemic, maternal, and neonatal outcomes [17,18], there is a lack of studies investigating the possible effects of an app on the diet of women with GDM. A systematic review has studied the usability of apps in the health care of pregnant women without GDM. That review indicated that apps may support women in reducing gestational weight gain and in increasing their intake of vegetables and fruits; however, the evidence of their effectiveness is still limited [19]. Dodd et al [20] evaluated the impact of a smartphone app as an adjunct to face-to-face consultation in facilitating dietary changes among pregnant women in South Australia. They found no significant benefit of the smartphone app in the intervention group. All women improved their dietary quality during pregnancy [20].

We have developed the Pregnant+ app for women with GDM [21]. This app provides tailored information on diet, physical activity, breastfeeding, and GDM, and the possibility to automatically transfer or manually record blood glucose levels from a glucometer to the smartphone (Multimedia Appendix 1 and Multimedia Appendix 2). The Pregnant+ app was developed in collaboration with experts in midwifery, obstetrics, physical activity, nutrition, and data security. Pregnant women with GDM of different ethnic origins were involved in several steps of its development [21]. A narrative review on studies with pregnancy-related apps found only 2 multilingual apps for use in prenatal care [22]. The Pregnant+ app is available in Norwegian, Urdu, and Somali languages. Information and pictures related to diet and physical activity are culturally adjusted according to the chosen language. This app was found to be the only app adapted to specific target groups (women born in Pakistan and Somalia) in a scoping review by Chen and Carbone [16]. The effect of this app on the main outcome (2-hour glucose level of the routine postpartum oral glucose tolerance test [OGTT]) was tested in a two-arm RCT at 5 diabetes outpatient clinics in the Oslo region, Norway [18,23]. The study showed that the Pregnant+ app did not have any significant effect on the main outcome [18].

The aim of this study was to examine if the Pregnant+ app had an effect on the dietary behavior of women. No specific dietary recommendation for women with GDM existed when the study was started in 2014. Women with GDM were recommended to follow the national dietary guidelines for healthy eating [24]. Some hospitals developed adjusted dietary advice for pregnant women with GDM. The Pregnant+ app promoted 10 GDM-specific dietary recommendations that were developed in cooperation with clinical nutritionists [21].

## Methods

### Study Design

We analyzed secondary data from a two-arm, multicentered, nonblinded RCT for women with GDM, which was conducted at 5 diabetes outpatient clinics in the Oslo region. This RCT is in accordance with the CONSORT-EHEALTH checklist (Multimedia Appendix 3).

### Recruitment

**Figure 1:**
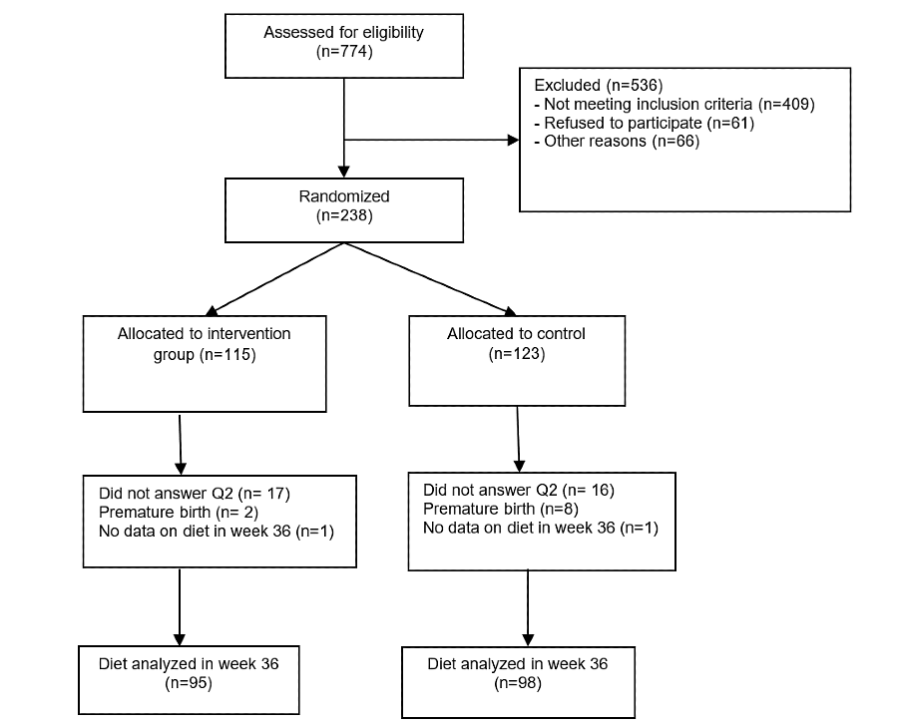
Flow chart describing the process leading up to the final number included in the analysis of dietary behavior in the Pregnant+ study.

Women with GDM were recruited from October 2015 to April 2017 by health care professionals at the diabetes outpatient clinics. At the time of recruitment, pregnant women with higher risk for GDM based on their prepregnancy weight, family history of diabetes, age, and ethnicity were sent for an OGTT [25]. Eligible women for this study were diagnosed with GDM by a 2-hour OGTT blood glucose level of ≥9 mmol/L, according to the definition of GDM in the Norwegian guidelines [25]. In addition, participants were older than 18 years, were less than 33-weeks pregnant, owned smartphones, and understood Norwegian, Urdu, or Somali. Women with type 1 or type 2 diabetes (OGTT blood glucose levels≥11 mmol/L), twin pregnancy, celiac disease, or lactose intolerance were excluded from the study. In total, 774 women were assessed for eligibility and 238 participated (Figure 1). Those who agreed to participate signed a consent form. All the participants in both groups received a glucometer and lancets from the study administrators. The Norwegian Social Science Data Services (identifier: 2014/38942) approved the study.

### Randomization and Blinding

The participants were randomly allocated to 2 groups: intervention (access to the Pregnant+ app and usual care) and control (usual care). Randomization was performed on a 1:1 basis with allocated blocks of 4. Women who agreed to participate filled out a baseline questionnaire (Q1) on an electronic tablet (average time 30-45 minutes). After completing Q1, a computer-based program randomized and allocated the women to either the intervention or the control group. The participating women, project workers, and health care professionals at the diabetes outpatient clinics were not blinded to the allocation.

### Intervention and Control

#### Usual Care (Control) Group

The participants in the control group received usual care for GDM according to the national guidelines [26]. This included regular consultations (every 1-2 weeks) with midwives or nurses, both specialized in diabetes, at the diabetes outpatient clinics. According to the guidelines [26], women should be provided with information about a healthy diet, with emphasis on regular meals with limited intake of sugar-rich foods and increased intake of whole grains and vegetables. The women in the control group were instructed how to measure their blood glucose levels and were asked to record these levels in a paper diary. They received written and verbal dietary advice on the basis of their blood glucose levels. In the absence of specific dietary guidelines for women with GDM, the different diabetes outpatient clinics included in the study developed some specific dietary guidelines for these women that emphasized regular meals, increased intake of vegetables and whole grains, and limited intake of sugar. If women in the usual care group downloaded the Pregnant+ app, their access was restricted to a single page with a link to the website of the Norwegian Directorate of Health with generic health information for women with GDM and a link to the Norwegian Federation of Diabetes.

#### Pregnant+ App and Usual Care (Intervention) Group

The participants in the intervention group had access to the Pregnant+ app in addition to usual care, as described above. The women allocated to the app group could download the app from the Apple Store or Google Play at the hospital or home. The app contained 4 main icons: “Blood glucose,” “Physical activity,” “Food and beverages,” and “Diabetes information.” The 10 GDM-specific dietary recommendations that were developed for this study [21] were presented in the “Food and beverages” icon for women (Textbox 1). The women could select if the dietary recommendations should be presented with food items and pictures representing the Norwegian, Urdu, or Somali food culture. They were also referred to recipes on the home page from the Norwegian Diabetes Foundation. They could automatically transfer or manually register their blood glucose levels in the icon “Blood glucose.” After registering, they received feedback on their values. Those with too high values were directly referred to the dietary recommendations. A graphical representation of the blood glucose levels visually aided the monitoring.

**The dietary recommendations in the Pregnant+ app.**

- Eat healthy meals regularly.
- Eat and drink little sugar.
- Eat more vegetables.
- Choose whole-grain products.
- Limit your intake of salt.
- Eat enough fish.
- Choose lean dairy milk produce.
- Choose healthy and less oil.
- Read nutrition labels on foods before buying.
- Choose water when thirsty.

### Measurements

The participants answered the questionnaires on an electronic tablet during their first consultation at a diabetes outpatient clinic and at their consultation around gestational week 36. The questionnaire included a 41-item food frequency questionnaire (FFQ). At baseline, they were asked to report their dietary habits prior to being diagnosed with GDM. In the second questionnaire, they were asked to report their current diet. The FFQ included the following food groups: beverages, milk and dairy products, bread and grain, fruit and vegetables, snacks, meat, and ready-to-eat meals. Answers to the questions on the frequency of intake ranged from 0 (never) to 9 (several times daily). The FFQ was based on the Fit for Delivery study and has been shown to have an adequate level of test-retest reliability [26]. The FFQs in Somali and Urdu were tested for comprehension and appropriateness by conducting qualitative interviews with Somali and Pakistani Norwegian women.

The healthy diet score for Pregnant+ (HDS-P+) was constructed using 9 subscales, with a possible range of 0 to 90. The subscales were constructed on the basis of the dietary recommendations (second point to tenth point) provided in Textbox 1 and consisted of different questions in the FFQ related to the dietary recommendations. The women were asked how often they choose different food groups, with the following answer options: 0=never, 1=less than once a week, 2=once a week, 3=twice, 4=three times, 5=four times, 6=five times, 7=six times, 8=every day, and 9=several times a day.

Information on background characteristics was obtained from the baseline questionnaire and consisted of different socioeconomic variables: age, education, income, country of birth, marital status, economic hardship, and language. Other variables related to pregnancy and health were parity, gestational age at baseline, prior GDM, and perceived health score [23].

### Statistical Analysis

Maternal baseline characteristics were compared according to randomization status. The characteristics were presented as mean (SD) for continuous variables (independent sample two-tailed *t* test) and proportions (%) for categorical variables (*χ*^2^ test). Dietary changes from baseline to around gestational week 36 were examined by a paired sample two-tailed *t* test. A one-way between-group analysis (analysis of covariance) was conducted to measure the effect of the Pregnancy+ app. The dietary behavior after the intervention was examined with adjustment to the baseline values. The HDS-P+ and subscales related to the recommended dietary advice were the dependent variables, and the randomization status (use of the Pregnant+ app or not) was the independent variable. Sensitivity analysis was performed to evaluate the effect of the differences in nonnative and native Norwegian-speaking women between the intervention and control groups at baseline. This did not alter the results and the final model did not adjust for this. Levene test and normality checks were carried out and the assumptions were met. All statistical analyses were performed with SPSS for IBM statistical software package (version 25, IBM Corporation). A two-sided *P* value of ≤.05 was considered significant.

#### Power

The power calculation was for the primary outcome for the RCT [23].

#### Data Exclusion

Figure 1 presents the flowchart for this study. Two women were excluded because of missing dietary data at gestational week 36. No other participant had more than 2 values missing in the 41-item FFQ. The missing data in this study were not imputed.

## Results

### Participant Characteristics

A total of 238 women were recruited at 5 diabetes outpatient clinics in the southeast region of Norway and randomized to use the Pregnant+ app (intervention group, n=115) or no app (control group, n=123). Figure 1 shows an overview of the final numbers in the dietary analysis. Of the 238 women, 193 (81.1%) women completed the FFQ both at baseline and at gestational week 36. Background characteristics are described according to the randomization status (Table 1). There were no significant baseline differences between the groups, except for more women with a non-Norwegian language as their first language in the intervention group (61 vs 46, *P*=.02).

**Table 1:** Background characteristics at baseline of the participants who provided dietary data at baseline (Q1) and after the intervention (Q2) in the study on the Pregnant+ app.

Background characteristics		Total, N=193	Control group, n=98	Intervention group, n=95	*P* value
**Age (years), n (%)**					.11
	≤29	47 (24.4)	22 (22)	25 (26)	
	30-37	110 (57.0)	52 (53)	58 (61)	
	≥38	36 (18.7)	24 (25)	12 (13)	
Gestational age at baseline, mean (SD)		27.1 (4.6)	27.3 (4.6)	26.9 (4.5)	.66
**Parity, n (%)**					.21
	Primiparous	86 (44.6)	48 (49)	38 (40)	
	Multiparous	107 (55.4)	50 (51)	57 (60)	
**Previous GDM^a^ (N=107^b^), n (%)**					.82
	No	75(70.1)	34 (68)	41 (72)	
	Yes	32 (29.9)	16 (32)	16 (28)	
**BMI (N=190^c^), n (%)**					.68
	<24.9	83 (43.7)	44 (45)	39 (42)	
	25.0-29.9	57 (30.0)	26 (27)	31 (33)	
	30.0-34.9	31 (16.3)	18 (19)	13 (14)	
	35.0-45.0	19 (10.0)	9 (9)	10 (11)	
**Country of birth, n (%)**					.15
	Norway	90 (46.6)	52 (53)	38 (40)	
	Western Europe + United States of America	13 (6.7)	9 (9)	4 (4)	
	Eastern Europe	18 (9.3)	9 (9)	9 (10)	
	Asia	45 (23.3)	16 (16)	29 (31)	
	Africa	22 (11.4)	10 (10)	12 (13)	
	South America	5 (2.6)	2 (2)	3 (3)	
**Marital status, n (%)**					.62
	Married/cohabiting	179 (92.7)	90 (92)	89 (94)	
	Single/other	14 (7.3)	8 (8)	6 (6)	
**Education, n (%)**					.51
	Primary school/no education	19 (9.8)	12 (12)	7 (7)	
	High school	40 (20.7)	23 (24)	17 (18)	
	College/university<4 years	47 (24.4)	23 (24)	24 (25)	
	College/university≥4 years	87 (45.1)	40 (41)	47 (50)	
**Smoking or wet tobacco** **, n (%)**					.69
	No	189 (97.9)	96 (98)	93 (98)	
	Yes	4 (2.1)	2 (2)	2 (2)	
**Main activity, n (%)**					.22
	Employed or self-employed	147 (76.2)	71 (72)	76 (80)	
	Not employed or not self-employed	46 (23.8)	27 (28)	19 (20)	
**Joined income, n (%)**					.78
	≤59,900 USD	57 (29.9)	26 (27)	31 (33)	
	60,000-79,900 USD	28 (14.2)	14 (14)	14 (15)	
	80,000-99,900 USD	39 (19.8)	20 (20)	19 (20)	
	≥100,000 USD	35 (18.8)	20 (20)	15 (16)	
	I don’t know	34 (17.3)	18 (18)	16 (17)	
**Economic hardship (N=188^c^), n (%)**					.85
	No	58 (30.9)	29 (30)	29 (32)	
	Yes	130 (69.1)	67 (70)	63 (69)	
**Language, n (%)**					.02
	Native Norwegian-speaking	86 (45.1)	52 (53)	34 (36)	
	Nonnative Norwegian-speaking	107 (54.9)	46 (47)	61 (64)	
Perceived health score (0**-**100), mean (SD)		70.8 (19.7)	70.5 (20.5)	71.2 (18.9)	.80

^a^GDM: gestational diabetes mellitus.

^b^Among multiparous women only.

^c^Some values are missing.

### Outcomes

#### Dietary Outcomes Around Gestational Week 36

Overall, the total HDS-P+ and most of the subscales, except the intake of healthy oils, improved from baseline to gestational week 36 (Table 2).

**Table 2:** Dietary changes from baseline to gestational week 36.^a^

Subscales		Baseline values, mean (SD)	Week 36 values, mean (SD)	*P* value
1. HDS-P+^b^		40.36 (14.11)	55.56 (13.70)	<.001
2. Sugar (times/week)		10.10 (7.88)	1.89 (3.21)	<.001
3. Vegetables (times/week)		8.87 (3.52)	10.35 (3.5)	<.001
4. Whole grains (times/week)		6.71 (2.96)	8.87 (2.78)	<.001
5. Salt (times/week)		3.71 (3.10)	2.39 (2.49)	<.001
6. Fish (times/week)		1.84 (1.17)	2.21 (1.32)	<.001
7. Low-fat milk (times/week)		4.84 (4.08)	4.22 (3.34)	.02
8. Healthy oil (% of total dietary fat)^c^		62.41 (25.32)	65.09 (25.36)	.11
9. Read nutrition labels		5.78 (3.44)	8.45 (2.64)	<.001
10. Water (% of total fluid intake)^c^		40.15 (14.67)	51.21 (17.35)	<.001

^a^Paired sample two-tailed *t* test.

^b^HDS-P+: healthy dietary score for Pregnant+.

^c^Percentage of weekly consumption.

#### Between-Group Differences After Intervention

A one-way between-group analysis of covariance was conducted to compare the effectiveness of the app on the participants’ dietary habits after being diagnosed with GDM. The women’s HDS-P+ preintervention was used as the covariate in the analysis. Table 3 presents the between-group differences for the overall HDS-P+ and the 9 different subscales at gestational week 36. No significant differences favored the intervention group. The analysis showed that the control group reported to eat more fish meals per week (*P*=.05). No other significant differences were found between the intervention and control groups. 

**Table 3:** Between-group differences in 10 dietary domains reported after the intervention (gestational week 36) in the Pregnant+ app.

Dietary domain	Subscale(s)	Control group, n=98, mean (SE)	Intervention group, n=95, mean (SE)	Estimated difference after intervention^a^, mean (SE)	95% CI	*P* value
Eat healthy	HDS-P+^b^	56.11 (1.11)	55.34 (1.13)	0.77 (1.72)	–2.62, 4.16	.65
Eat and drink little sugar	Sugar (times/week)	1.97 (0.31)	1.79 (0.32)	0.18 (0.45)	–0.70, 1.06	.68
Eat more vegetables	Vegetables (times/week)	10.40 (0.32)	10.30 (0.32)	0.09 (0.45)	–0.83, 0.95	.86
Choose whole grains	Whole grains (times/week)	8.23 (0.27)	8.01 (0.28)	0.14 (0.40)	–0.79, 0.99	.73
Limit your intake of salt	Salt (times/week)	2.53 (0.12)	2.25 (0.21)	0.28 (0.31)	–0.31, 0.88	.35
Eat enough fish	Fish (times/week)	2.34 (0.09)	2.09 (0.09)	0.26 (0.13)	–0.01, 0.51	.05
Choose lean dairy milk	Low-fat milk (times/week)	4.40 (0.28)	4.04 (2.85)	0.35 (0.40)	–0.42, 1.14	.38
Eat less saturated fat	Healthy oil (% of total dietary fat)^c^	66.30 (2.05)	63.80 (2.10)	2.50 (2.50)	–3.31, 8.33	.40
Read nutrition labels	Read labels	8.76 (0.25)	8.13 (0.25)	0.63 (0.36)	–0.07, 1.33	.08
Choose water	Water (% of total fluid intake) ^c^	51.93 (1.57)	50.47 (1.59)	1.46 (2.24)	–2.69, 5.89	.57

^a^Analysis of covariance adjusted for baseline HDS-P+.

^b^HDS-P+: healthy dietary score for Pregnant+.

^c^Percentage of weekly consumption.

## Discussion

### Principal Results

The Pregnant+ app combined with usual care did not have any significant effect on the dietary behavior of the participants during pregnancy compared to the dietary behavior of the participants receiving usual care only. All the participants improved their diet from the time they were diagnosed with GDM to gestational week 36.

### Comparison With Prior Work

This study adds to the literature on the development and effect of pregnancy-related apps for the management of GDM and for following a healthy diet [22,27,28]. Pregnant women consider these apps to be useful and convenient for nutrition information and management of their diets [27]; however, little is known about their effects on the dietary behavior [19]. mHealth apps may provide several functions targeting behavior change or monitoring. The most successful smartphone-based interventions for dietary change and health outcomes include elements of self-monitoring and personalized feedback [29]. Similar to other apps for women with GDM [21,30], the Pregnant+ app includes a function for self-monitoring of blood glucose levels. According to our qualitative study on women’s experience with the Pregnant+ app, the self-management of blood glucose levels was the most important aspect of the app for increasing self-awareness and motivation [31]. Ten of the 17 participants from the intervention group reported to use the Pregnant+ app daily for their blood glucose management. However, the monitoring of food intake was not possible in the app. A qualitative study about the acceptability of a smartphone app for patients with type 2 diabetes indicated that the use of a digital diabetes diary to monitor food intake supported them in eating a healthy diet [32]. Dodd et al [20] assessed the effect of a smartphone app on the dietary behavior of pregnant women. Their app included a combination of information provision, goal setting, feedback, and self-monitoring. The use of the app was poor, and it provided no additional benefit over face-to-face consultation and printed materials in improving dietary behaviors [20]. It should also be considered that adherence to self-monitoring has been shown to decrease over time and that self-monitoring is successful only when people are regularly reminded to use the app [33]. The women in our intervention group were not reminded to use the app.

Compared to studies demonstrating the positive impact of apps on healthy eating and blood glucose levels [29,34,35], the Pregnant+ app did not have any personal interaction with the women. For instance, a review on the use of telemedicine technology for managing diabetes in pregnancy (not just GDM) showed a modest but statistically significant improvement in HbA_1c_ levels [35]. To avoid bias, the health care professionals were not asked to use the app actively during their consultations. However, the women automatically received specific dietary information when registering too high blood glucose levels in the Pregnant+ app. The women in our qualitative study wanted more involvement of the health care professionals in the usage of the app. In an RCT involving 203 pregnant women in the United Kingdom, women with access to a smartphone app had a higher level of satisfaction with care than the women in the control group [17]. In this study, the midwives checked the women’s registered blood glucose levels in the app 3 times a week and sent feedback via SMS text messaging [17].

Similar to that reported in other mHealth studies [36], technical problems with the app could be a reason for us to not find any effect of the app on the participants’ diets. Some of them experienced problems with the automatic transfer of the blood glucose levels [31]. A cross-sectional survey on the use of mHealth among Latino patients with diabetes found that the lack of operability between the smartphone app and other devices could serve as a barrier to using the app [37].

All the participants in this study improved their diet after being diagnosed with GDM. This is in accordance with previous research, indicating that the diagnosis of GDM motivates women to change their diets [38-40]. Our previous study about women’s dietary habits prior to being diagnosed with GDM showed low adherence to national dietary recommendations [41]. A significantly higher proportion of nonnative Norwegian-speaking women had a high healthy diet score compared with native Norwegian-speaking women. In this study, significantly more women with a non-Norwegian language as their first language were in the intervention group (*P*=.02). Previous research has shown that a combination of technological, health literacy, and language issues may result in a lower uptake of pregnancy apps among immigrant women [16]. These barriers may also have led to a lower usage of the Pregnant+ app.

### Limitations

One of the main limitations of this study was that we did not have access to usage logs because of technical problems. To secure the participants’ privacy, we did not collect any additional data from the app. We do not know if those in the intervention group actually used the Pregnant+ app or about their frequency of usage or the pages in the app accessed by them. Our qualitative study on 17 participants from the intervention group showed that some women used the app regularly and some did not use it at all because of technical problems [31]. Patients participating in a study will often experience an effect even when not receiving the intervention—the Hawthorne effect. In our study, this could account for the lack of effects as women not using the app may have focused more on a healthy diet as a result of participating in a study. The data for this study were derived from self-completed questionnaires, which include the possibility for recall bias. Social desirability might have biased the self-report of dietary intake. The FFQ covered only selected aspects of the overall diet. Thus, the difference in the HDS-P+ should not be interpreted as an absolute measure of dietary change [42].

### Conclusions

To our knowledge, this is one of the first studies to evaluate the effect of a smartphone app on the dietary behavior of women with GDM [17]. Our findings do not support the supplementation of face-to-face follow-up of women with GDM with a smartphone app in the presence of high-standard usual care. However, the app might be a useful tool for women who do not receive sufficient dietary counselling in person. Future research should explore the effects of various technological features provided in a smartphone app to improve the care of women with GDM.

## Appendix

Multimedia Appendix 1 Screenshot of the app.

Multimedia Appendix 2 Screenshot of the dietary recommendation in the app.

Multimedia Appendix 3 CONSORT-EHEALTH Checklist (V 1.6.1).

## Abbreviations

FFQ: food frequency questionnaire
GDM: gestational diabetes mellitus
HDS-P+: healthy diet score for Pregnant+
mHealth: mobile health
OGTT: oral glucose tolerance test
RCT: randomized controlled trial
