# Supplementary figures and images for: Effect of the Pregnant+ Smartphone App on the Dietary Behavior of Women With Gestational Diabetes Mellitus: Secondary Analysis of a Randomized Controlled Trial
Source: JMIR Mhealth Uhealth. 2020 Nov 4;8(11):e18614. doi: 10.2196/18614 (PMC7673980; doi:10.2196/18614)

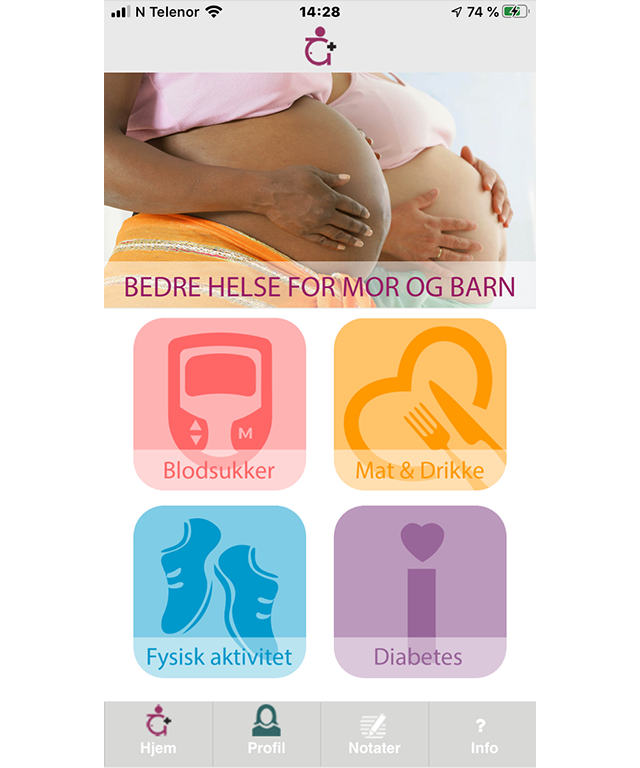

Supplement: Multimedia Appendix 1 [file mhealth_v8i11e18614_app1.png]

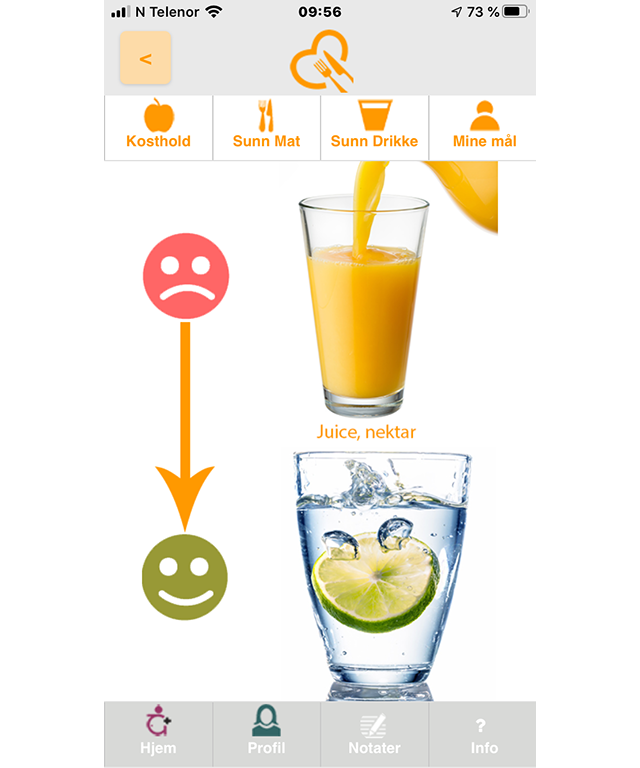

Supplement: Multimedia Appendix 2 [file mhealth_v8i11e18614_app2.png]
